# Archive for Research in Child Health (ARCH) and Baby Gut: Study Protocol for a Remote, Prospective, Longitudinal Pregnancy and Birth Cohort to Address Microbiota Development and Child Health

**DOI:** 10.3390/mps4030052

**Published:** 2021-08-03

**Authors:** Eliot N. Haddad, Sarah S. Comstock

**Affiliations:** Department of Food Science and Human Nutrition, Michigan State University, East Lansing, MI 48824, USA; haddadel@msu.edu

**Keywords:** gut microbiome, pregnancy, human milk exposure, child development, infancy, microbiota, cohort

## Abstract

The infant gut microbiome is shaped by numerous factors such as diet and the maternal microbiota and is also associated with later atopy and obesity. The Archive for Research in Child Health and Baby Gut (ARCHBG) cohort was established in 2015 to (1) understand how the development of the infant gut microbiota is associated with atopy, obesity, and gastrointestinal disease and (2) characterize the associations of maternal pre-pregnancy BMI and infant diet with the development of the gut microbiota. Study participants for ARCHBG are convenience samples recruited through two pipelines in Lansing and Traverse City, Michigan: (1) Archive for Research in Child Health (ARCH_GUT_) and (2) BABY_GUT_. A total of (*n* = 51) mother–infant dyads have been enrolled to date. This prospective cohort study collects maternal pre-pregnancy fecal samples, maternal data, child fecal samples at four timepoints (one week, six months, 12 months, and 24 months), and child data up to five years of age. All samples and data are collected remotely by mail, phone, or drop-off at select locations. Of all participants enrolled, 76.5% (n = 39) of infants have a complete record of stool samples. At least 88.2% (n = 45) of fecal samples were submitted at each timepoint. ARCHBG will allow for a nuanced understanding of the temporal development of the infant gut microbiome and numerous child health outcomes.

## 1. Introduction

The human gut microbiota is a key factor in perinatal research that is associated with numerous pregnancy and infant factors such as mode of delivery, diet, antibiotic use, adiposity, and human milk exposure [1,2]. In spite of this, logistic and pragmatic ethical considerations often prevent adequate experimental analysis to determine causal ties between the gut microbiome and numerous human health outcomes [3]. Hence, prospective, longitudinal, cohort studies are valuable tools to ascertain the directionality of the relationships and associations of the gut microbiome in pregnant women and their infants to child health outcomes.

Recently, the gut microbiome has also been demonstrated to play an important role in the development of diseases from infancy into childhood [4]. With a lifetime asthma prevalence of 12.7% in Americans [5], and with the significant increase in the incidence of allergies [6], it is imperative to develop a nuanced understanding of the corollary pathogenesis associated with altered gut microbial conditions in infancy, a time when the immune system is still developing. For example, antibiotic-induced dysbiosis of the infant gut microbiome has been associated with a greater risk of later developing asthma, obesity, and intestinal bowel disease [7,8,9]. Further research is needed to identify the pathways for these associations in light of the numerous confounding variables involved in infant growth and health.

The ARCH_GUT_/BABY_GUT_ (ARCHBG) study is a convenience sample of mother/infant dyads from the Lansing and Traverse City areas of Michigan. This prospective, longitudinal pregnancy and birth cohort study began in 2015, and recruitment into the BABY_GUT_ cohort arm is ongoing. The design of the research methodologies used for this cohort serve as an important template for the research community to conduct remote sample collection and maintain participant engagement through incentivized follow-up participation. Both of these design factors are relevant not only to the pandemic of today, but also to busy families during any moment in time as well as to the continued efforts of implementing cost-effective strategies to conduct the research of tomorrow.

Primarily, the objective of the ARCHBG cohort study is to comprehend how the long-term development of the gut microbiota within infants through preadolescence is associated with food allergies, asthma, and other atopic diseases, in addition to obesity. Secondary objectives include characterization of maternal pre-pregnancy body mass index (BMI) and infant dietary associations with the gut microbiota and the development of gastrointestinal (GI) diseases. Recently, it has been suggested that the initial seeding of the infant microbiome may occur in utero, but that the major event of microbial colonization of the infant is during birth [10]. Indeed, infants born vaginally exhibit distinct gut microbiomes from those born by C-section, and exhibit less risk for developing asthma and obesity in later life [11]. The longitudinal data that has been collected and continues to be collected from ARCHBG will aid efforts by researchers to characterize the complex gut microbial relationships corresponding to the development of atopy, obesity, and gastrointestinal disease, and the impact of results from this cohort can be expanded through merging data with other cohort studies of sufficiently similar design.

## 2. Design and Population

Study participants for ARCHBG are convenience samples recruited through two separate pipelines. The first, ARCH_GUT_, enrolled pregnant women as a subgroup of the Archive for Research in Child Health (ARCH) pregnancy cohort [12]. ARCH is designed to be low-cost, with medical records, birth certificates, newborn blood spots, blood samples, and urine samples all collected at the time of routine clinical check-ups, with no extra burden on participants. ARCH_GUT_ was an optional extension of ARCH focused on collecting questionnaire responses and fecal samples (maternal third trimester of pregnancy; infant at one week, six months, 12 months, and 24 months) from participants who provided written, informed consent. ARCH_GUT_ enrollment occurred between 2015–2017 in a clinic in Lansing, Michigan and another in Traverse City, Michigan, with n = 29 mothers enrolling at the time of pregnancy.

The BABY_GUT_ arm was developed as a supplement to ARCH_GUT_. Using the same overall methods, participants were recruited at several clinics in Lansing, Michigan. The clinics were afforded pamphlets describing the study. Incentives included personal (monetary) and societal (understanding atopy, obesity and gastrointestinal disease) benefits to participating in the study. Prospective participants interested in enrolling called the phone number provided in the pamphlet, and after providing written, informed consent, were successfully enrolled in the study. BABY_GUT_ commenced in 2016 and is ongoing, with n = 22 participants successfully enrolled to date.

**Figure 1:**
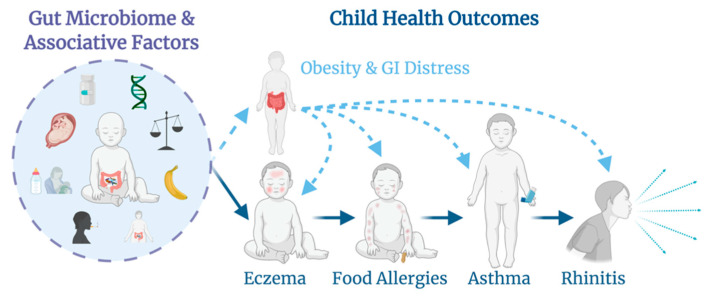
The ARCHBG cohort is designed to study the main exposures associated with the development of the gut microbiome in infancy and how the microbiota development relates to the outcomes of obesity, atopy, and gastrointestinal disease.

ARCHBG is open to all English-speaking, 18+ pregnant women and their infants. As of August 2020, the total enrolled population of ARCHBG is N = 51, with potential for continued growth and a target sample of n = 100. The study collects information from both the mother and infant that allows for gut microbial analysis in light of numerous health exposures, such as diet and maternal pre-pregnancy BMI, and outcomes such as the development of atopy, obesity, or GI diseases (Figure 1). The longitudinal nature of this cohort study allows for a temporal analysis of the onset of atopy or obesity and the development of the gut microbiome over time. ARCHBG is an ongoing cohort study, and apart from initial enrollment, which may be done via mail or in person, participant data and samples are collected remotely through the use of telephones, postal service, or drop-off at select locations. Both ARCH_GUT_ and BABY_GUT_ were approved by the Michigan State University Human Research Protection Program (IRB C07-1201 (ARCH), 15–1240 (BABY_GUT_) and 14–170M (ARCH_GUT_)).

## 3. Procedures

### 3.1. Data Collection

After participants are successfully enrolled in ARCHBG, they complete an enrollment questionnaire and provide fecal samples during their third trimester of pregnancy (Table 1). The enrollment questionnaire surveys the parents’ health statuses and any suspected/diagnosed allergies they may have [13]. It also asks about body weight, height, antibiotic use, smoking, age, intention to breastfeed, size of family household, and various demographic factors (race, ethnicity, education, marriage, and income/assets). Along with the fecal sample at the third trimester of pregnancy, participants also complete a sample questionnaire which requests additional information about proximal probiotic/prebiotic/antibiotic use, whether the mother is currently sick, water source, and a recall of food/drink consumed in the past 24 h. Upon return of the maternal fecal sample, participants are rewarded with either a baby box (containing diapers, a mattress, a onesie, a sleeper, a blanket, a hat, and mittens) or a $25 gift card for a local store.

Mothers are also expected to return infant fecal samples at one week, six months, 12 months, and 24 months post-partum. At each of these timepoints and until five years (with potential for up to 12 years follow-up upon provision of consent), questionnaires are administered regarding various health parameters (Table 2). At one week and six months, questionnaires collect data about infant medicinal use, mother’s medicinal use, whether the infant is sick, human milk exposure at the breast in the past 24 h, human milk fed from a bottle in the past 24 h, formula exposure in past 24 h, formula name/brand, other foods ingested, prebiotic/probiotic use, infant weight, infant length, mode of delivery, infant sex, and whether the infant attends a care center or is cared for outside of the home. Starting at six months, questionnaires also assess all food/drink consumed in past 24 h. From 12 months onward, the questionnaire expands to account for suspected allergies, diagnosed medical conditions, and breastfeeding continuation/cessation in addition to child height/weight, childcare in home or outside home, and antibiotic use in the past year. Questionnaire data is collected either by mail or by phone call according to the participant’s preference, ensuring a streamlined, low-burden process. Recently, questionnaires administered through a web-based survey software (Qualtrics, Provo, UT) have also been offered for the three-, four-, and five-year follow-up periods.

All fecal samples are collected by the participants themselves with Para Pak collection tubes (Meridian Biosciences, Cincinnati, OH, USA). At each collection timepoint, participants are sent kits containing handouts on proper collection procedures for both the maternal (third trimester) and infant fecal samples. Overall, the maternal kit contains a stool hat, a Para Pak collection tube, gloves, a small zip-locking bag, paper towels, a water/tear-resistant envelope, a stamped return box, and the sample info questionnaire (Figure S1a). Baby sample kits contain diapers in place of the stool hat (Figure S1b). Fecal samples are mailed to the lab or picked up from a participant-designated location, aliquoted in the lab, and stored in a −80 °C freezer until further processing. For each infant fecal sample returned, participants are given a $10 gift card for a local store. From the one- year timepoint on, participants are mailed a children’s book each year that they respond to the data questionnaire. Participants also receive a $10 gift card to a local store for completion of the 4- and 5-year questionnaires.

### 3.2. Statistical Analysis

Aims of the statistical analyses for ARCHBG are to (1) observe the development and exposures associated with gastrointestinal pathologies, obesity, and atopy and (2) determine gut microbial differences between participants and identify the underlying, associated exposures. The exact statistical methods and processes will vary according to the project and its specifications, but there are commonalities to the approach. Demographic differences between populations that differ by a variable of interest will be compared using t-tests for parametric and Wilcoxon rank-sum tests for nonparametric continuous data. Categorical variables are typically compared using chi-squared tests. BMI and human milk exposure measures are grouped into categorical units to ensure adequate statistical power. Measures of microbial diversity are calculated in R [14]. Alpha diversity, or within-sample variation in number of individual taxa and their respective abundances (richness and evenness), is measured using Chao1, Shannon, and Inverse Simpson indices through the vegan package [15]. Significant differences between alpha diversity indices are assessed with ANOVA for parametric data, with normality confirmed using the Shapiro–Wilk test. If nonparametric, alpha diversity indices are assessed with the Wilcoxon rank-sum test or Kruskal–Wallis test depending on number of groups being compared. Power calculations for alpha diversity are conducted using G*power version 3.1.9.2 [16]. Beta diversity, or differences in microbial community structure and composition between samples, is visualized using principal coordinates analysis (PCoA) for Sorensen or Bray–Curtis dissimilarity scores. Significant differences between group means and variances are tested using permutational multivariate analysis of variance (PERMANOVA) and permutation of dispersion (PERMDISP) from the adonis and betadisper functions, respectively, in the vegan package. Power calculations for beta diversity are conducted using the micropower package [17]. Taxa that differ significantly between groups are tested using a negative binomial model from the MASS package [18]. *p*-values less than 0.05 are considered significant, with the Benjamini–Hochberg procedure applied for false discovery rate correction.

## 4. Results

### 4.1. Population Characteristics

**Figure 2:**
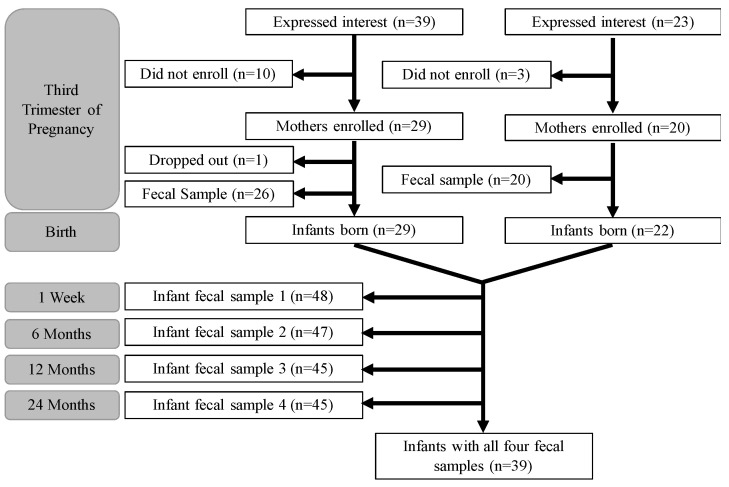
Participant flowchart of mothers and infants in ARCHBG.

From the overarching ARCH study, n = 39 prospective mothers initially expressed interest in participating in ARCH_GUT_. Of these, n = 29 provided written, informed consent and completed an enrollment form. One participant dropped out, leaving n = 28 total participants. One mother had twins, accounting for a total of n = 29 mother–infant dyads from the ARCH_GUT_ recruitment branch. For BABY_GUT_, n = 23 prospective mothers expressed initial interest, with n = 20 enrolling. Of these, n = 2 mothers enrolled two infants in the study, accounting for n = 22 mother–infant dyads from BABY_GUT_. This leaves a total of n = 51 participants who have enrolled in ARCHBG to date. Of these, n = 39 infants have a complete record of stool samples, with at least n = 45 (88.2%) fecal samples at each timepoint (Figure 2).

The ARCH_GUT_ and BABY_GUT_ cohorts did not statistically differ except in stock/bond ownership (Table 3). There were no statistically significant differences between participants who submitted all fecal samples versus those who missed at least one infant/maternal fecal sample (Table S1).

### 4.2. Maternal Pre-Pregnancy BMI and the Pregnancy/Early Infant Gut Microbiome

Analysis of maternal pre-pregnancy BMI with the maternal fecal microbiome in the third trimester of pregnancy revealed lower gut microbial diversity among overweight mothers compared to normal or obese mothers (Chao1, *p* = 0.02; Inverse Simpson, *p* = 0.05; Shannon, *p* = 0.02). However, there were no significant differences in alpha diversity among one-week infant fecal samples after stratifying groups based on maternal BMI (Chao1, *p* = 0.58; Inverse Simpson, *p* = 0.49; Shannon, *p* = 0.51). Univariate analysis revealed significant differences in infant beta diversity community composition (Sorensen) arising from breastmilk exposure (*p* = 0.008) and mode of delivery (*p* = 0.001). Infants consuming breastmilk exclusively were significantly enriched in *Escherichia–Shigella* and *Staphylococcus* and lacking in *Akkermansia* and *Acidaminococcus* when compared to infants consuming a mixed diet (formula and breast milk). Additionally, infants with obese mothers were significantly enriched in *Akkermansia* and *Acidaminococcus* compared to infants with either overweight or normal-weight mothers. These results delineate associations of maternal pre-pregnancy BMI with both maternal and early infant gut microbial parameters [19].

### 4.3. Human Milk Exposure and Six-Month Infant Gut Microbial Diversity

At six months [20], human milk exposure (categorized into 100%, 80%, 50–80%, and <20% groups based on estimated fraction in infant diet) within the last 24 h was significantly different for infants based on their mother’s pre-pregnancy BMI category (Chi-Square, *p* = 0.02), with all normal weight mothers exposing their infants to 50%+ human milk in their diets compared to approximately one-third of overweight mothers and two-thirds of obese mothers exposing their infants to less than 20% human milk in their diets. Infants consuming less than 20% human milk in their diets had significantly higher alpha diversity in comparison to all other human milk exposure groups (Chao1, *p* = 0.01; Inverse Simpson, *p* < 0.001; Shannon, *p* < 0.001), a finding which is corroborated by existing literature [21]. Infant gut microbial community structures (Bray–Curtis) differed significantly between individuals in different human milk exposure categories (Genus: *p* = 0.01; Phylum: *p* = 0.005) and by maternal BMI category (Genus: *p* = 0.03; Phylum: *p* = 0.03). Multivariable linear regression models accounting for human milk exposure, maternal BMI category, sample shipping time, age, sex, delivery mode, and cohort demonstrated that human milk exposure consistently and significantly accounted for the most variance in beta diversity genus richness (Sorensen) with *p* ≤ 0.002 and R^2^~0.1 for all models. *Megasphaera*, unclassified *Lachnospiraceae,* and *Blautia* were all significantly lower for infants consuming 100% breast milk at six months of age.

### 4.4. Fecal Resistome in Pregnancy and Six-Month Infants

After screening 321 antibiotic resistance genes (ARG) and 53 mobile genetic elements (MGE) in maternal (pregnancy) and six-month infant fecal samples, 133 genes were found in at least one sample and therefore used in the analysis. Genes conferring resistance to tetracycline and the related transposon sequences were most abundant among all samples. Tetracycline resistance genes were also the genes most commonly shared between mother–infant dyads, with 50% sharing the same ARG. On average, 29% of ARG and 24% of MGE in the fecal samples were shared between mothers and their infants, but infant MGE compositions were more similar between unrelated infants than between each infant and their own mother (Sorensen; *p* = 0.014). Infant fecal samples also had more MGE but no significant differences in the number of ARGs in comparison to the pregnancy samples. The diversity of ARGs and MGEs was significantly higher in infants than in the mothers (ARG, *p* = 0.016; MGE, *p* = 0.0036). The multi-drug resistance, sulfonamide, and fluoroquinolone ARGs were all significantly (*p* < 0.001) enriched in infant samples compared to pregnancy samples, which were significantly (*p* < 0.001) enriched in the aminoglycoside, vancomycin, and macrolide-lincosamide-streptogramin B ARGs. ARG patterns for those consuming >50% breastmilk were significantly different compared to those consuming less (Bray–Curtis, *p* = 0.043). Overall abundance patterns of ARGs and gut microbial taxa were associated (Procrustes, *p* = 0.001). However, there were no significant associations between the infant fecal resistome and antibiotic exposure in pregnancy or postpartum [22].

### 4.5. Infant Growth and the Gut Microbiota at 12 Months

Human milk exposure at six and 12 months of infant age differed significantly by pre-pregnancy maternal BMI category (*p* = 0.01). All infants with mothers in the normal BMI range had some proximal human milk exposure, but 36.4% of infants with overweight mothers and 66.7% of infants with obese mothers were reported to have no proximal exposure to human milk at the time of fecal sampling when six or 12 months of age. Infants exposed to human milk had significantly lower gut microbial richness than infants who were not exposed (Chao1, *p* = 0.01). Although maternal pre-pregnancy BMI was associated with significantly greater infant gut microbial diversity (Chao1, *p* = 0.01; Shannon, *p* = 0.04), a bivariate analysis revealed that human milk exposure was the likely driver of this observed association (Chao1, *p* = 0.03; Shannon, *p* = 0.07), and not maternal BMI (Chao1, *p* = 0.49; Shannon, *p* = 0.54). At 12 months of age, infant BMI-for-age *z*-scores were only significantly related to maternal pre-pregnancy BMI (*p* = 0.01), and not human milk exposure (*p* = 0.53) or the infant gut microbiota (Chao1, *p* = 0.95; Shannon, *p* = 0.10; Bray–Curtis, *p* = 0.90; Sorensen, *p* = 1) [23].

## 5. Discussion

### 5.1. Principle Findings

ARCHBG is a pregnancy cohort that allows for continued remote follow-up and the collection of fecal samples from birth until two years of age and data throughout the growth of an infant until a maximum of 12 years. This cohort provides the opportunity to study gut microbial associations with various developmental milestones, such as growth, dietary transitions, and, notably, the development of various gastrointestinal and atopic maladies. The data that has been collected starting in 2015 has allowed for several analyses delineating associative factors with the maternal gut microbiome, the early infant gut microbiome, antibiotic resistance, human milk exposure, and infant growth. The lack of significant differences between the ARCH_GUT_ and BABY_GUT_ cohorts, save for stock/bond ownership, justifies combining these two foundational cohorts to form the overall ARCHBG cohort (Table 3). The median shipping time was four days at each sample collection time point, and the high rate of infant fecal sample collection at each timepoint indicates a high rate of retention of our participants, with n = 39 (76.5%) submitting all four infant fecal samples and a minimum submission rate of n = 45 (88.2%) at each timepoint (Figure 2). Although this is a high rate of response and retention, it serves to emphasize the quintessential issue of participant attrition in longitudinal cohorts.

Especially in the age of COVID-19, studies requiring high-levels of participatory involvement are increasingly difficult to conduct due to social reluctance and legislative restrictions on in-person gatherings aimed at slowing the spread of the disease [24]. These obstacles add to an already difficult domain of data collection and longitudinal engagement in perinatal research, leading to the requisite consideration of novel research methods to overcome these barriers [25].

### 5.2. Strengths of the Study

As a prospective cohort study, ARCHBG has the potential to provide quality data regarding the temporal progression of various conditions in conjunction with the development of the gut microbiome. The collection of both maternal (pregnancy) and four infant fecal samples at different timepoints allows for the examination of familial versus environmental associations, and can prevent recall bias for confounders arising from pregnancy, a common issue in cohort studies [26]. This is especially important in light of emerging evidence that the pregnancy gut microbiome is involved in fetal development and health outcomes [27]. Many cohort studies do not collect maternal fecal samples [27,28,29,30,31,32,33,34,35,36,37,38,39]. For those that do, maternal fecal samples are usually collected at birth or post-partum [40,41,42,43,44,45,46,47,48], with very few cohorts collecting fecal samples during gestation [49]. Additionally, the ARCHBG questionnaires recorded both maternal and paternal health history and conditions, which provides a more complete picture than maternal data alone on the potential etiology of future conditions within the growing infant [50].

The salient strength of the design of ARCHBG is the remote methodology utilized for the collection of samples and data. The only potential physical contact between the mother and researchers is at enrollment, with all later interaction occurring by mail, telephone, or pick-up from a designated location. Due to the current COVID-19 pandemic, the study design of ARCHBG is especially relevant as it provides a template for future research that addresses the common challenges associated with perinatal research that have been compounded amidst a global health crisis, especially among vulnerable populations [51]. These challenges include participant attrition, labor-dependence for interviewer-administered questionnaires and sample collection, high costs, and logistical obstacles for the participation of mothers and their infants [50,52,53]. Although recruitment occurred at a health clinic, the ability to remotely engage in the research may encourage women who would otherwise have been lost to follow-up due to missed appointments, transportation issues, or relocation to a new city. Furthermore, it may appease those families with skepticism of modern healthcare, and it provides the ability for women to discuss with their partners before choosing to participate [25]. Although collecting questionnaires and fecal samples by mail is a technique that has been around for several decades, the postal questionnaires used in this study were designed with best practice recommendations in mind, ensuring financial incentives for fecal samples, a concise, personal questionnaire, providing stamped return envelopes, and following-up with participants post-contact [54]. This likely contributed to the relatively low attrition rates for a pregnancy cohort requiring repeated engagement.

Considering that the US median household income was $68,703 in 2019 [55] and that n = 20 (43.5%) of our cohort report earning a household income of less than $50,000 annually (Table 3), ARCHBG is also more representative of the national spectrum of socioeconomic statuses than other cohorts, which are often skewed towards families with higher incomes [29].

### 5.3. Limitations of the Data

The pivotal limitation thus far to ARCHBG is the recruitment of participants which has fallen below anticipated levels. This presents issues in the generalizability of the data due to low power for some associations with small effects and limited sample size. As a convenience sample of volunteers, costs associated with recruitment have been kept to a minimum. However, a more involved, provider-based sampling model, while clearly more expensive and resource-intensive, may be worth exploring in future studies due to its ability to enroll a high percentage of eligible and geographically-representative women [56].

Participant recruitment is also limited to Traverse City and Lansing, MI. In the future, expanding the overall recruitment zones for ARCHBG may provide a more comprehensive view of gut microbial development in Michigan infants. Additionally, the vast majority of participants elected to complete the questionnaires on paper and return by mail. However, we do not collect information on perceived burden of each mode of questionnaire collection (mail, phone, internet), so it is difficult to ascertain which mode is most efficient for participants.

Though the mailing of fecal samples eases participatory burdens and research-associated costs, it also can affect the quality of microbial data, as the sample is subjected to less-than-ideal conditions during the mailing period. Although this results in changes to the microbial data compared to samples that are immediately frozen [57], gut microbial differences between samples are preserved in spite of short-term storage at room temperature, conserving result validity [58]. In addition, the ARCHBG protocol only includes 16S rRNA amplicon metagenomics. In the future, studies may benefit from applying other ‘omics’ analyses to the fecal samples to obtain a greater depth and breadth of information. Furthermore, the remote nature of ARCHBG calls for self-reported measurements for parameters such as breastmilk exposure and growth metrics, but these have also been demonstrated to be valid among maternal populations [59,60].

Finally, despite relatively low attrition rates among participants (Figure 2) and no significant differences between those who submitted all fecal samples versus those who missed at least one (Table S1), there is always room to further improve non-response and dropout rates to limit potential selection bias. Attrition may be reduced in the future by incorporating web-based questionnaires, which has been shown in a randomized intervention follow-up to be effective in garnering a high level of data retention (~89%) alongside time-dependent bonus incentives [61]. Internet-based methodologies will also further reduce costs and may be no more prone to selection bias than traditional follow-up methodologies [62]. Considering the reach and cost-effectiveness of web-based strategies, future pregnancy cohorts may be well-advised to complement both their recruitment and follow-up methodologies with online options.

## 6. Conclusions

The design of ARCHBG provides a valuable template for future research that examines the gut microbiome or seeks to remotely engage participants over an extended period of time. Despite the low number of participants in the initial analyses, the preliminary results provide insight into important factors associated with the infant gut microbiome, be they maternal BMI, infant diet, or infant growth, that merit further examination as more participants are recruited and enrolled. ARCHBG welcomes collaboration with other qualified research teams seeking to examine covariates within the dataset or to combine data with that of a similar pregnancy cohort for more robust analysis. Apart from the current research directions and preliminary findings, ARCHBG is anticipated to contribute to the growing body of knowledge centering on maternal–infant wellbeing, with implications for policy and practical life.

## Figures and Tables

**Table 1:** Overview of maternal data and samples collected during pregnancy. ^a^

Informed Consent
Enrollment Questionnaire
Suspected/diagnosed allergies—father
Suspected/diagnosed allergies—mother
Maternal birth year
Pre-pregnancy maternal weight
Pregnancy maternal weight
Maternal height
Antibiotic use in past year
Smoking exposure
Parity
Intention to breastfeed
**Maternal Fecal Sample & Sample Questionnaire**
Current antibiotic use
Current health status (sick/not sick)
24-h dietary recall
Water source
**Demographic Survey**
Race
Ethnicity
Education level
Marital status
Household income
Stocks/bond ownership
Car ownership
Home ownership

^a^ Each bolded phrase indicates a data collection point with the information collected at that point in time indicated in normal font below the bolded heading.

**Table 2:** Data and sample collection schedule for children in the ARCHBG cohort study.

	1 Week	6 Months	12 Months	24 Months	3–5 Years
Infant/Child Fecal Sample	✓	✓	✓	✓	
**Infant/Child Sample Questionnaire ^a^**	✓	✓	✓	✓	✓
Infant birth date	✓				
Infant birth weight	✓				
Infant birth length	✓				
Current medication use	✓	✓	✓	✓	
Prior medication use	✓	✓	✓	✓	
Maternal medication use (30 days pre-birth)	✓				
Baby health status (sick/not sick)	✓	✓	✓	✓	
24-h proximal human milk exposure	✓	✓	✓	✓	
24-h proximal formula exposure (name and brand)	✓	✓	✓	✓	
24-h proximal diet	✓	✓	✓	✓	
Estimation of percentage of infant diet that is human milk	✓				
Prebiotic/probiotic	✓	✓	✓	✓	✓
Avoided foods			✓	✓	✓
Infant health problems		✓	✓	✓	✓
Any symptoms after specific foods			✓	✓	✓
Presentation of allergic symptoms			✓	✓	✓
Diagnosis of symptoms			✓	✓	✓
Have you visited a doctor or ER because of symptoms?			✓	✓	✓
How long after exposure until you experience the symptom?			✓	✓	✓
Diagnosed health conditions			✓	✓	✓
Breastfeeding length			✓	✓	✓
Formula feeding length			✓	✓	✓
Solid food introduction		✓	✓	✓	✓
Child weight		✓	✓	✓	✓
Child height		✓	✓	✓	✓
Concerns about body weight			✓	✓	✓
Location of childcare			✓	✓	✓
Medical procedures performed on child			✓	✓	✓
Antibiotic use in past year			✓	✓	✓
Exposure to smoke			✓	✓	✓

^a^ Variables collected on the questionnaire are indicated below. Each variable collected is indicated by a checkmark in the box below the time point of questionnaire administration. Some information is collected once, whereas other information is collected repeatedly.

**Table 3:** Sociodemographic information and sample shipping time for ARCHBG participants.

	ARCH_GUT_	BABY_GUT_	Overall (ARCHBG)
**Infant**			
N	29	22	51
Female ^b^	10 (34.5)	6 (27.3)	16 (31.4)
**Mother**			
N	28	20	48
Race ^b^			
*White/Caucasian* ^b^	21 (75.0)	17 (85.0)	38 (79.2)
*Black/African American* ^b^	4 (14.3)	2 (10.0)	6 (12.5)
*Other* ^b^	3 (10.7)	1 (5.00)	4 (8.33)
Age at Birth (years) ^c^	30.9 ± 3.7	31.7 ± 5.1 *	31.2 ± 4.3 *
College Degree ^b^	16 (59.3) *	14 (70.0)	30 (63.8) *
Married ^b^	21 (75.0)	17 (85.0)	38 (79.2)
Household Income ≥ 50,000 USD ^b^	14 (51.9) *	12 (63.2) *	26 (56.5) **
Own a Home ^b^	11 (39.3)	11 (55.0)	22 (45.8)
Own Stocks/Bonds ^a,b^	9 (32.1)	14 (70.0)	23 (47.9)
Own a Car ^b^	26 (92.9)	20 (100)	46 (95.8)
**Sample Shipping Time (days)**			
Pregnancy (3rd Trimester) ^d^	4 (1–7); 24	4 (0–11); 18	4 (0–11); 42
One Week Infant ^d^	4 (2–14); 24	4 (0–11); 20	4 (0–14); 44
Six Month Infant ^d^	5 (1–22); 23	3 (0–9); 21	4 (0–22); 44
12 Month Infant ^d^	4 (1–11); 23	3.5 (2–31); 20	4 (1–31); 43
24 Month Infant ^d^	4 (1–12); 21	4 (0–14); 18	4 (0–14); 39

* Each asterisk indicates a missing data point. ^a^ Statistically significant difference (*p* < 0.05) between ARCH_GUT_ and BABY_GUT_. ^b^ n (%). ^c^ mean ± standard deviation. ^d^ median (range); n. Bolded phrases indicate subcategories of information. Race categories are indicated in italics.

## Supplementary Materials

The following are available online at https://www.mdpi.com/article/10.3390/mps4030052/s1, Figure S1, Photo of (a) maternal fecal sample collection kit and (b) infant fecal sample collection kit., Table S1, Comparison of sociodemographic information and sample shipping time for ARCHBG participants who submitted all fecal samples versus those that missed at least one fecal sample.
