# Supplementary material for: Archive for Research in Child Health (ARCH) and Baby Gut: Study Protocol for a Remote, Prospective, Longitudinal Pregnancy and Birth Cohort to Address Microbiota Development and Child Health
Source: Methods Protoc. 2021 Aug 3;4(3):52. doi: 10.3390/mps4030052 (PMC8395764; doi:10.3390/mps4030052)
Supplement: Supplementary file 1 [file mps-04-00052-s001.zip › mps-1266544-supplementary.pdf]

Table S1. Comparison of sociodemographic information and sample shipping time for ARCHBG participants who submitted all fecal samples versus those who missed at least one fecal sample.

	**Submitted All Infant and Maternal Samples**	**Missed Infant or Maternal Sample**	**Overall (ARCHBG)**
**Infant**			
N	37	14	51
Female [a]	11 (29.7)	5 (35.7)	16 (31.4)
**Mother**			
N	35	13	48
Race [a]			
White/Caucasian [a]	28 (80.0)	10 (76.9)	38 (79.2)
Black/African American [a]	4 (11.4)	2 (15.4)	6 (12.5)
Other [a]	3 (8.57)	1 (7.69)	4 (8.33)
Age at Birth (years) [b]	31.0 ± 4.4 *	31.1 ± 3.8	31.2 ± 4.3 *
College Degree [a]	24 (70.6) *	6 (46.2)	30 (63.8) *
Married [a]	30 (85.7)	8 (61.5)	38 (79.2)
Household Income ≥ 50,000 USD [a]	14 (42.4) **	12 (92.3)	26 (56.5) **
Own a Home [a]	18 (51.4)	4 (30.8)	22 (45.8)
Own Stocks/Bond [a]	19 (54.3)	4 (30.8)	23 (47.9)
Own a Car [a]	35 (100)	11 (84.6)	46 (95.8)
**Sample Shipping Time (days)**			
Pregnancy (3rd Trimester) [c]	4 (0–11); 32	3.5 (0–7); 10	4 (0–11); 42
One Week Infant [c]	4 (0–14); 35	4 (2–9); 9	4 (0–14); 44
Six Month Infant [c]	4 (0–22); 34	3 (1–12); 10	4 (0–22); 44
12 Month Infant [c]	4 (1–31); 36	2 (2–9); 7	4 (1–31); 43
24 Month Infant [c]	4 (0–14); 35	3.5 (1–8); 4	4 (0–14); 39

* Each asterisk indicates a missing data point. Therefore, if there are two asterisks, there are two missing data points.

[a] n (%)

[b] mean ± standard deviation

[c] median (range); n

No statistically significant differences.

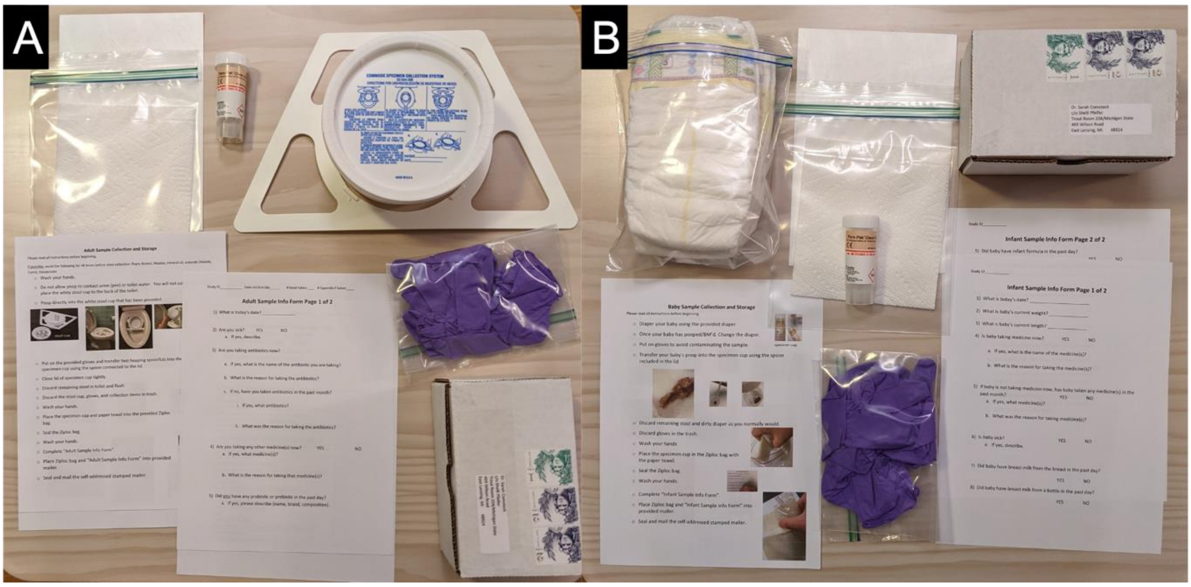

Figure S1. Photo of (**a**) maternal fecal sample collection kit and (**b**) infant fecal sample collection kit.
